# The extent of population genetic subdivision differs among four co-distributed shark species in the Indo-Australian archipelago

**DOI:** 10.1186/1471-2148-9-40

**Published:** 2009-02-12

**Authors:** Jenny R Ovenden, Tom Kashiwagi, Damien Broderick, Jenny Giles, John Salini

**Affiliations:** 1 Molecular Fisheries Laboratory, Department of Primary Industries and Fisheries, Queensland Government, PO Box 6097, St Lucia, Queensland 4067, Australia; 2 School of Biomedical Sciences, University of Queensland, Queensland 4072, Australia; 3 School of Integrative Biology, University of Queensland, Queensland 4072, Australia; 4 CSIRO Marine Research, PO Box 120, Cleveland, Queensland 4163, Australia

## Abstract

**Background:**

The territorial fishing zones of Australia and Indonesia are contiguous to the north of Australia in the Timor and Arafura Seas and in the Indian Ocean to the north of Christmas Island. The area surrounding the shared boundary consists of a variety of bio-diverse marine habitats including shallow continental shelf waters, oceanic trenches and numerous offshore islands. Both countries exploit a variety of fisheries species, including whaler (*Carcharhinus spp*.) and hammerhead sharks (*Sphyrna spp*.). Despite their differences in social and financial arrangements, the two countries are motivated to develop complementary co-management practices to achieve resource sustainability. An essential starting point is knowledge of the degree of population subdivision, and hence fisheries stock status, in exploited species.

**Results:**

Populations of four commercially harvested shark species (*Carcharhinus obscurus*, *Carcharhinus sorrah*, *Prionace glauca*, *Sphyrna lewini*) were sampled from northern Australia and central Indonesia. Neutral genetic markers (mitochondrial DNA control region sequence and allelic variation at co-dominant microsatellite loci) revealed genetic subdivision between Australian and Indonesian populations of *C. sorrah*. Further research is needed to address the possibility of genetic subdivision among *C. obscurus *populations. There was no evidence of genetic subdivision for *P. glauca *and *S. lewini *populations, but the sampling represented a relatively small part of their distributional range. For these species, more detailed analyses of population genetic structure is recommended in the future.

**Conclusion:**

Cooperative management between Australia and Indonesia is the best option at present for *P. glauca *and *S. lewini*, while *C. sorrah *and *C. obscurus *should be managed independently. On-going research on these and other exploited shark and ray species is strongly recommended. Biological and ecological similarity between species may not be a predictor of population genetic structure, so species-specific studies are recommended to provide new data to assist with sustainable fisheries management.

## Background

The Indo-Australian archipelago is a biogeograhically complex region encompassing a series of continental shelves, volcanic mountainous islands and deep-sea trenches. The Indonesian section straddles the equator and extends about 5000 km from east to west. It has the world's highest marine endemism; including most the diverse sea-grass meadows, greatest expanses of mangroves and most extensive coral reef communities [1]. The Australian section is dominated by an extensive continental shelf extending offshore from three Australian state jurisdictions; Queensland, Northern Territory and Western Australia. As the world's fourth most populous nation, Indonesia has a high demand for food from the sea. The landed tonnage of Indonesian fisheries is orders of magnitude larger than Australian fisheries. Indonesian and Australian fishing zones are contiguous along much of the northern Australian Exclusive Economic Zone that extends 200 km from shore. There is considerable goodwill between the countries for cooperative management of shared fisheries stocks [2].

The Indo-Australian archipelago contains about 30% of the one thousand shark and ray species in the world [3]. Many of the species are endemic and new species of sharks and rays continue to be described [eg. [4]]. Indonesia has the world's largest elasmobranch catch [118,000 t in 2003, 1], but data on catch and effort in the commercial and artisanal fisheries is severely lacking. In Australia, the elasmobranch catch was approximately 3000 tonnes in 2001 consisting of about 180 species as target and incidental (bycatch) catch [5]. Only seven of these species are regarded as adequately managed and monitored, with the remainder being data-deficient for the purposes of assessing the sustainability of current catch rates [6]. Faced with minimal species-specific information, fisheries managers have to generalise across biologically similar groups when making predictions about the effects of fisheries on shark and ray populations.

Population genetic analysis at and below the species level is an important starting point for species conservation and sustainable exploitation. The biological unit described by fisheries mathematical population models (*sensu *stock assessment) is self-maintained by reproduction and subsequent recruitment and experiences natural and human-mediated mortality. Individuals within the unit share the same gene-pool and are genetically isolated from other such groups [7]. Population genetic analyses can identify the number of such management units within the geographical distribution of a target species [8]. Population genetic analyses can also identify groups of populations that represent a significant part of the evolutionary legacy of the species, which may have a higher conservation status than other populations in the range of the species [9]. Other methods, such as otolith microchemical analyses [10] and analyses of parasite distribution and abundance [11] can detect population groupings below the species level that may exist in response to, or in addition to, genetic differentiation between populations.

The aim of this study was to assess intra-specific population genetic subdivision for four shark species that are co-distributed in the Indo-Pacific and that support fisheries in northern Australia and Indonesia; Dusky Shark *Carcharhinus obscurus*, Spot-tail Shark *Carcharhinus sorrah*, Blue Shark *Prionace glauca *(Fa. Carcharhinidae) and Scalloped Hammerhead *Sphyrna lewini *(Fa. Sphyrnidae). Carcharhinids and Sphyrnids are placental livebearers with low intrinsic rates of increase. *Prionace glauca *and *S. lewini*, however, have notably higher fecundities than *C. sorrah *and *C. obscurus *[12]. All are predators with naturally low abundances. Except *C. sorrah*, the study species have worldwide distributions; *C. obscurus *is found in coastal and offshore, but not oceanic, waters; *P. glauca *is oceanic but may be found close inshore when the continental shelf is narrow and *S. lewini *is semi-oceanic occurring over continental and insular shelves and adjacent deep water [13]. *Carcharhinus sorrah *is restricted to the tropical Indo-west Pacific and is found on continental and insular shelves, primarily near reefs. The IUCN Red List of Threatened Species (Global) lists *C. obscurus*, *P. glauca *and *S. lewini *as 'near threatened' and *C. sorrah *as data deficient.

We expected that the extent of genetic subdivision between shark populations would be low because of their capacity to move extensively within the geographic constraints of the study area. Three of the four species (*C. obscurus*, *P. glauca*, *S. lewini*) attain large body sizes [greater than 3.0 m; *C. sorrah *are smaller, 1.5–3.0 m; 12]. All species are regarded as strong and active swimmers, but this may not lead to dispersal. The average distance moved was 50 km for 23 species of sharks tagged and recaptured off the northern Australia coastline between 1983 and 1997 [14], although the maximum distance moved was greater than 1100 km. Dispersal occurs during juvenile to adult phases, as there is no larval stage. Genetic subdivision has been demonstrated for *S. lewini*, but it was reported on a larger spatial scale than our study area [eg. between Pacific and Atlantic Ocean basins, [15]]. Genetic subdivision between populations was observed for another *Carcharhinus *species (*C. limbatus*) linked to the occurrence of female philopatry to nursery areas in the Atlantic coast of the US [16]. There are few studies on elasmobranch population structure in the Indo-west Pacific. Dudgeon et al [17] reports population subdivision in the leopard shark (*Stegastoma fasciatum*) in Australia and south-east Asia, however this species is demersal and its capacity for dispersal is unknown. Barriers to dispersal in the Indo-Pacific could include deep-sea trenches and strong currents. Various phylogenetic 'breaks' for marine species from sea-horses to mackerel have been proposed [18-21]. The results are discussed as the type of the information needed by management agencies for sustainable harvesting plans of elasmobranchs in the bio-diverse Indo-Australian region.

## Methods

### Collection locations and DNA extraction

**Figure 1:**
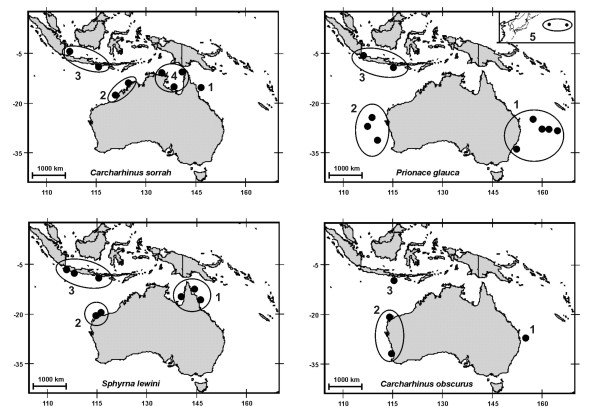
**Circles encompass collection locations for four shark species from East Australia (1), West Australia (2), Indonesia (3), Gulf of Carpentaria (4) and mid-north Pacific (5)**.

Tissue samples from *C. obscurus*, *C. sorrah*, *P. glauca *and *S. lewini *were collected from two areas in Australia and one area in central Indonesia (Fig. 1). An additional Australian location was included from the Gulf of Carpentaria for *C. sorrah *to provide another test of gene flow restriction between northern Australia and Indonesia. A more distant location (mid-north Pacific) was included for *P. glauca *to test the spatial extent of genetic homogeneity (Fig. 1). The mtDNA sequence of one sample of *S. lewini *from the Atlantic Ocean was included as a link between this study and that of Duncan et al [15].

In Australia, sharks were sampled from commercial catches by on-board observers and fisheries biologists. In Indonesia, samples were taken from landed catch at local markets (Muara Angke, western Java and Tanjung Luar, Lombok) by fisheries biologists. The fishing grounds represented were assumed to be within a 100 – 300 km radius of the markets. White muscle (~200 mg) was sampled from each specimen and preserved in either DMSO solution (20% dimethylsulphoxide in saturated NaCl solution) or 70% ethanol solution.

Total genomic DNA was extracted from ten to 50 mg of preserved tissue using the Chelex method [22]. After heat denaturation and brief centrifugation (1200 g for 5 min), the supernatant containing genomic DNA was removed to a fresh tube for subsequent manipulation and storage.

Taxonomic field identifications were based on morphological features, verified with photographs where necessary. While identification of the four species in this study is relatively unproblematic for Carcharhinid and Sphyrnid sharks, samples lacking photo verification were confirmed by comparing mtDNA sequence to a reference data-base for Carcharhiniformes species maintained by JO's research group and by principal coordinates analysis of genetic distance using GenAlex 6.1 [23] between all pairs of samples within a species based on their microsatellite genotype. Outliers and misidentified samples were removed from the study.

### Mitochondrial DNA methods – laboratory

The 5' end of the control region (CR) of the mitochondrial DNA (mtDNA) was amplified and sequenced. Approximately 1145 bp (base pairs) were amplified using the forward primers ProL2 (5' CTGCCCTTGGCTCCCAAAGC 3') and the reverse primer PheCacaH2 (5' CTTAGCATCTTCAGTGCCAT 3') designed by Pardini et al. [24]. Each 50 μl PCR reaction contained 1× PCR buffer, 200 μM of each dNTP, 1 μM of each primer, 2.5 mM of MgCl_2_, 4 units of Taq DNA polymerase (Qiagen, Doncaster, Vic, Australia) and 5 μl of DNA template. The PCR cycling conditions were 90 s at 94°C followed by 35 cycles of 5 s at 94°C, 30 s at 50°C and 30 s at 72°C with a final extension of 72°C for 5 min. Amplified DNA was purified using a commercial purification kit. The cycle sequencing reaction used ABI Big Dye Terminators v3.1^®^. Approximately 400 bp of the control region was sequenced in one direction using the forward primer. Sequences were obtained using an automated sequencer (Applied Biosystems 3130xl).

### Microsatellite methods – laboratory

Shark samples were genotyped with three to five microsatellite loci (*C. obscurus*, four loci; *C. sorrah*, five loci; *P. glauca*, five loci and *S. lewini*, three loci). Microsatellite loci were sourced from Ovenden et al [25], Feldheim et al [26] and Keeney and Heist [27] and were generally applied to non-target species (ie. as cross-species amplifications). Microsatellite PCRs were performed in 96-well plates using a Perkin Elmer 9700 thermocycler. Reactions (10 μl) contained 1 μl of PCR buffer ^® ^(Qiagen P/L, Doncaster, Vic, Australia) containing Tris-HCl (pH 8.7), KCl and (NH_4_)_2 _SO4; 4 mM MgCl_2_; 0.02 μM forward primer with an M13 extension [28,29]; 0.2 μM reverse primer; 1.9 μM fluoro-labeled M13 primer; 0.3 units *Taq *DNA polymerase (Qiagen); 200 μM dNTP (Pharmacia Biotech, GE Healthcare Bio-Sciences P/L, Rydalmere, Australia); 1% bovine serum albumin and approximately 25 ng genomic DNA template. The DNA template and enzyme were denatured at 94°C for 1 min 30 s, followed by 35 cycles consisting of 94°C for 5 s, 60°C for 20 s and 72°C for 30 s and a final extension at 72°C for 30 min. Loci were amplified in separate reactions and then combined for fragment separation according to label colour and fragment size. Microsatellite fragment separation and scoring was performed using capillary electrophoresis (ABI3130xl). The size in base pairs of microsatellite amplicons was calculated to two decimal places and amplicons were allocated to a 'bin' that represented the mean allele size.

### Mitochondrial DNA methods – data analyses

MtDNA control region sequence data was edited, aligned and checked by eye using the software Sequencher v 4.1 (Genecodes, Ann Arbor, MI, US). Haplotypes and numbers of variable sites for each species were determined using MacClade v3.08. Haplotype and nucleotide diversities were calculated using Arlequin version 3.11 [30]. Intraspecific phylogenies among haplotypes were estimated using Bayesian [MrBayes v 3.1, 31], maximum likelihood (ML) and parsimony methods [PAUP* v 4.0b10, 32]. Candidate species for use as outgroups were evaluated on the availability of CR sequence, ease of sequence alignment, interspecies genetic similarity based on cytochrome oxidase I sequence [33] and robustness of topologies to alternate combinations of outgroups. For Bayesian and ML analyses, the most appropriate model of DNA evolution was selected using Akaike Information Criteria using ModelTest v 3.7 [34]. The General Time Reversible (GTR) model with gamma distributed among-site rate variation was preferred for *P. glauca *(gamma 0.608). The GTR was also preferred for *C. sorrah *with equal rates of variation among sites. The Hasegawa Kishino Yano 85 model (HYK85) was preferred for *C. obscurus *and *S. lewini*. Gamma was 0.479 for *S. lewini *and zero for *C. obscurus*. ML phylogenies were estimated using PAUP* v 4.0b10 [32] and nodal support was assessed using non-parametric bootstrapping involving 2000 pseudo-replicates of the original sequence alignment. Bayesian phylogenetic analysis was implemented using MrBayes v 3.1 [31]. Four MCMC chains were run, starting from different random trees that were sampled every 100 generations. Each MCMC run consisted of one million generations after which the average standard deviation of split frequencies was less than 0.01 and the potential scale reduction factor was reasonably close to 1.0 for all parameters, as recommended by the MrBayes manual. The first 25% percent of generations were discarded as burn in. The posterior probability values, after burn in and across the four MCMC runs, were used to construct a 50% majority rule consensus phylogeny. Intraspecific hapotype networks were constructed using the statistical parsimony method of Templeton et al [35] with a 95% connection limit using the TCS v 1.21 software [36].

The proportion of total mtDNA CR sequence variation that was due to genetic differentiation between populations (pairwise Φ_ST_) was measured for the one Indonesian and two Australian populations. Where available, additional populations were included in the pairwise analyses. Tamura-Nei distances [37] were used to describe sequence variation between haplotypes, with gamma values determined by ModelTest v 3.7 (above). The significance of Φ_ST _values was tested by comparing the real Φ_ST _with the Φ_ST _values produced from 1023 random permutations of the data using Arlequin version 3.11 [30].

### Microsatellite methods – data analyses

The null hypothesis of Hardy-Weinberg equilibrium was tested using Genepop v 4.0.7 [38]. The program Micro-checker [39] was used to investigate likely causes for possible deviation from Hardy-Weinberg equilibrium. Microsatellite genetic diversity was characterised by the number of alleles per locus, expected (H_E_) and unbiased (UH_E_) heterozygosity, observed heterozygosity (H_O_) and fixation index using GenAlex 6.1 [23]. The probability of rejecting the null hypothesis of genotypic disequilibrium between pairs of loci across populations was tested using Genepop v 4.0.7 [38]. Bonferroni corrections to *p*-values for performing multiple tests was determined by the method of Sankoh et al [40]. Microsatellite allelic diversity was used to investigate the degree of genetic subdivision between Australia and Indonesia for the four shark species. This was done using a standard population-pairwise F_ST _approach [41] implemented in GenAlex v 6.1 [23] and Genepop v 4.0.7 [38]. Non-parametric bootstrapping was implemented in GenAlex v6.1 to estimate *p*-values over 999 randomizations of the data set. Missing data for population pair-wise comparisons was handled by interpolation in GenAlex v6.1.

## Results

### Carcharhinus obscurus

**Figure 2:**
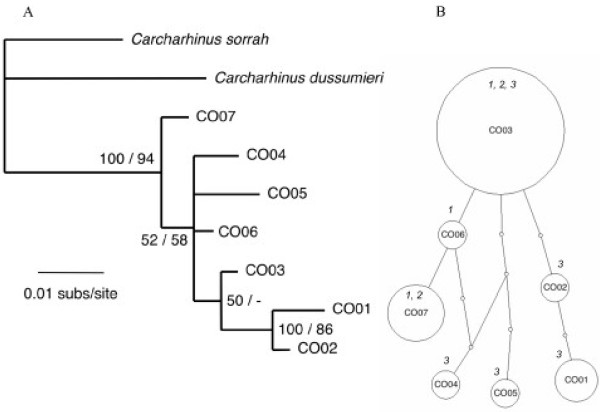
**Inferred phylogeny (A) and statistical parsimony network (B) among haplotypes of *C. obscurus***. The phylogeny was rooted with *C. sorrah *and *C. dussumieri *and nodal support is given as Bayesian posterior probabilities/ML boostrap support. Dash (-) indicates support of less than 50%. In the network, each indicated step (circle) represents a single nucleotide difference in the mtDNA control region sequence. The area of circles is scaled to represent the relative frequency of that haplotype and the smallest circle represent inferred haplotypes that were not sampled. The collection location of sampled haplotypes is numbered (in italics) according to Fig. 1.

Seven *C. obscurus *mtDNA haplotypes were found from 28 individuals sequenced. Haplotype frequencies varied among collection locations. For instance, haplotype CO7 was found in 15% of the West Australia samples and 28% of the samples from East Australia, but was not found in Indonesia (Table 1). Nucleotide and haplotype diversity also varied among locations. Both measures were highest in Indonesia and lowest in the two Australian locations (Table 1). Two haplotypes, which were only found in Indonesia (CO01 and CO02), were placed in a well-supported clade (Bayesian posterior probability, 100; ML bootstrap, 86), but other haplotypes only found in Indonesia (CO4, CO5) were not part of this clade (Fig. 2). There was evidence that haplotypes CO01 and CO02 formed a distinct lineage within a parsimony network of haplotypes (Fig. 2).

**Table 1:** Control region mtDNA haplotypes (with numbered polymorphic sites), haplotype frequencies, shared haplotypes and indices of population diversity for *Carcharhinus obscurus*.

*C. obscurus *haplotypes (375 bases) Total N = 28													
Haplotype	4	6	1	1	1	2	3	3	3	3	Indonesia (n = 8)	West Australia (n = 13)	East Australia (n = 7)
	5	3	1	7	9	0	0	1	1	2			
			8	5	3	0	9	2	3	2			

CO1	A	T	G	T	A	C	A	A	T	T	0.25	-	-
CO2	.	.	.	C	G	.	.	.	.	.	0.125	-	-
CO3	.	.	A	C	G	T	.	.	.	.	0.375	0.846	0.571
CO4	.	C	A	C	G	T	G	G	.	.	0.125	-	-
CO5	.	.	A	C	G	T	G	.	A	C	0.125	-	-
CO6	.	C	A	C	G	T	.	.	.	.	-	-	0.143
CO7	G	C	A	C	G	T	.	.	.	.	-	0.154	0.286
Number of haplotypes											5	2	3
Number of polymorphic sites											10	2	2
Nucleotide diversity per location (within population, %)											1.044 ± 0.666	0.160 ± 0.153	0.297 ± >0.250
Haplotype diversity per location (within population)											0.857 ± 0.108	0.282 ± 0.142	0.667 ± 0.160

Key: Haplotype CO1 has Genbank accession number EF363709.

*Carcharhinus obscurus *microsatellite genotypes were obtained from four loci. Low numbers of microsatellite genotypes were obtained from East Australia, which had downstream analysis implications. For that population, small sample sizes increased the confidence limits around allele frequencies [42] and most likely caused disequilibrium in genotype proportions at two of the four loci (Table 2). One of these loci (CS02) was out of HW equilibrium among samples from other locations. The mean unbiased expected heterozygosity across populations and loci was 0.762. The mean number of alleles across populations was four for locus Cli108, 10.67 ± 3.33 (mean ± SE) for locus CS02, 13.33 ± 2.67 for locus CS06 and 3.33 ± 0.33 for locus LS24. Genotypic disequilibrium was not detected for pairwise comparisons of the three loci (ie. excluding CS02) in the three populations.

**Table 2:** The population, sample size (N), number of microsatellite alleles per locus (Na), average observed heterozygosity (Ho) and expected (He) and unbiased (UHe) heterozygosity and fixation index (F) for each sampling location for *Carcharhinus obscurus*.

*C. obscurus*								
	Locus	N	Na	Ho	He	UHe	F	Signif
Indonesia	Cli108	22	4	0.73	0.69	0.71	-0.05	
	CS02	18	14	0.56	0.88	0.91	0.37	*
	CS06	17	16	0.88	0.86	0.88	-0.03	
	LS24	25	3	0.44	0.52	0.53	0.15	
West Australia	Cli108	66	4	0.58	0.59	0.59	0.02	
	CS02	48	15	0.42	0.90	0.91	0.56	*
	CS06	27	16	0.89	0.88	0.89	-0.01	
	LS24	59	4	0.54	0.56	0.56	0.03	
East Australia	Cli108	5	4	0.80	0.70	0.78	-0.14	
	CS02	4	4	0.50	0.72	0.82	0.30	*
	CS06	5	8	1.00	0.86	0.96	-0.16	
	LS24	7	3	0.14	0.56	0.60	0.75	*

Key: Significant deviations from Hardy-Weinberg equilibrium are shown (*).

Among *C. obscurus *collected from Indonesia and West Australia, genetic population subdivision was found using mtDNA control region sequence (Φ_ST _0.191), but not microsatellite allele frequencies (Table 3). This did not change when the analysis was repeated without the locus that was not in Hardy-Weinberg proportions (CS02). Comparisons involving East Australia across the two classes of genetic markers were not significant.

**Table 3:** Statistically significant microsatellite pairwise F-statistics (below diagonal) and mtDNA Φ_ST _(above diagonal) for populations of *Carcharhinus obscurus*.

*C. obscurus*			
	Indonesia	West Australia	East Australia
Indonesia	-	0.191	NS
West Australia	NS	-	NS
East Australia	NS	NS	-

Key: F-statistics were significant using an experiment wide alpha of 0.05 after adjusting for multiple comparisons via a sequential Bonferroni correction. NS indicates non-significance. There was no change to population pairwise F_ST_'s from microsatellite data when locus CS02 was removed.

### Carcharhinus sorrah

**Figure 3:**
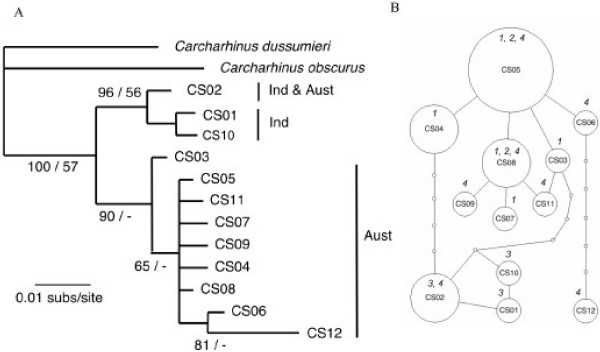
**Inferred phylogeny (A) and statistical parsimony network (B) among haplotypes of *C. sorrah *collected in Indonesia (Ind) and Australia (Aust)**. The phylogeny was rooted with *C. obscurus *and *C. dussumieri *and nodal support is given as Bayesian posterior probabilities/ML boostrap support. Dash (-) indicates support of less than 50%. In the network, each indicated step (circle) represents a single nucleotide difference in the mtDNA control region sequence. The area of circles is scaled to represent the relative frequency of that haplotype and the smallest circle represent inferred haplotypes that were not sampled. The collection location of sampled haplotypes is numbered (in italics) according to Fig. 1.

Twelve mtDNA haplotypes were found for *C. sorrah*, and there were pronounced differences in their frequencies between the three collection locations. Two haplotypes (CS01 and CS10) were only found in Indonesia, nine haplotypes were only found in Australian samples and only haplotype (CS02) was shared between Indonesia and Australia (Table 4). Phylogenetic analysis separated the Australian and Indonesian sequences into two well-supported clades (Fig. 3). The haplotype that was shared between Australia and Indonesia (CS02) showed close similarity to the Indonesian group of haplotypes. A single shark with haplotype CS02 was collected from Australian waters (Gulf of Carpentaria) suggesting that it, or its maternal ancestor, may have dispersed from Indonesia. Haplotypes found in Indonesia (CS01, CS02 and CS10) formed a cluster on the parsimony network and their connection to the remainder was equally parsimonious via either CS03 or CS04.

**Table 4:** Control region mtDNA haplotypes (with numbered polymorphic sites), haplotype frequencies, shared haplotypes and indices of population diversity for *Carcharhinus sorrah*.

*C. sorrah *haplotypes (375 bases) Total N = 49																				
Haplotype	3	4	5	5	1	1	1	1	2	2	2	3	3	3	3	3	Indonesia (n = 8)	West Australia (n = 8)	East Australia (n = 13)	Gulf of Carpentaria (n = 20)
	9	5	0	6	2	3	4	7	3	3	9	0	1	1	2	5				
					0	9	9	8	1	7	8	9	0	4	0	2				

CS01	T	G	T	C	A	C	A	A	T	A	T	A	T	G	A	C	0.250	-	-	-
CS02	.	.	.	A	.	.	.	.	.	.	.	.	.	.	.	.	0.500	-	-	0.050
CS03	.	.	C	A	G	.	G	.	C	.	C	.	.	.	.	.	-	-	0.077	-
CS04	.	.	C	A	G	T	G	.	C	.	.	.	.	.	.	.	-	-	0.385	-
CS05	.	.	C	A	G	T	G	.	C	.	C	.	.	.	.	.	-	0.875	0.308	0.600
CS06	.	A	C	A	G	T	G	.	C	.	C	.	.	.	.	.	-	-	-	0.050
CS07	C	.	C	A	G	T	G	G	C	.	C	.	.	.	.	.	-	-	0.077	-
CS08	.	.	C	A	G	T	G	G	C	.	C	.	.	.	.	.	-	0.125	0.154	0.100
CS09	.	.	C	A	G	T	G	G	C	G	C	.	.	.	.	.	-	-	-	0.100
CS10	.	.	.	.	.	.	.	.	.	.	C	.	.	.	.	.	0.250	-	-	-
CS11	.	.	C	A	G	.	G	G	C	.	C	.	.	.	.	.	-	-	-	0.050
CS12	.	A	C	A	G	T	G	.	C	.	C	G	C	C	G	T	-	-	-	0.050
Number of haplotypes																	3	2	5	7
Number of polymorphic sites																	2	1	4	14
Nucleotide diversity per site (within population, %)																	0.267 ± 0.228	0.067 ± 0.095	0.324 ± 0.247	0.535 ± 0.351
Haplotype diversity per location (within population)																	0.714 ± 0.123	0.250 ± 0.180	0.782 ± 0.079	0.642 ± 0.118

Key: Haplotype CS01 has Genbank accession number FJ161688.

*Carcharhinus sorrah *samples were assayed in sufficient numbers (43.3 ± 2.6, mean ± SE) for statistical analyses from four populations using five microsatellite loci (Table 5). There were three instances of genotypic proportions not following Hardy-Weinberg expectations, but as they were not associated with particular loci or collection locations, their effect on subsequent analyses were judged to be slight. Unbiassed heterozygosity was 0.55 ± 0.07 and the number of alleles was 3.50 ± 0.96 for locus LS15, 12.50 ± 1.66 for locus CT05, 6.25 ± 0.18 for locus CS12, 5.50 ± 0.96 for locus Cli100 and 26.25 ± 1.44 for locus CS08 across loci and populations. Genotypic disequilibrium was not detected for pairwise comparisons of the five loci in the four populations.

**Table 5:** The population, sample size (N), number of microsatellite alleles per locus (Na), average observed heterozygosity (Ho) and expected (He) and unbiased (UHe) heterozygosity and fixation index (F) for each sampling location for *Carcharhinus sorrah*.

*C. sorrah*								
	Locus	N	Na	Ho	He	UHE	F	Signif
Indonesia	LS15	31	2	0.03	0.03	0.03	-0.02	
	CT05	25	10	0.76	0.79	0.81	0.04	
	CS12	47	5	0.47	0.55	0.56	0.15	
	Cli100	42	4	0.05	0.07	0.07	0.32	*
	CS08	28	24	0.86	0.94	0.96	0.09	
West Australia	LS15	53	4	0.09	0.13	0.13	0.25	
	CT05	51	17	0.92	0.89	0.90	-0.04	
	CS12	51	7	0.61	0.57	0.57	-0.07	
	Cli100	46	8	0.50	0.45	0.46	-0.11	
	CS08	40	27	0.98	0.95	0.96	-0.02	
East Australia	LS15	37	2	0.08	0.08	0.08	-0.04	
	CT05	37	10	0.89	0.83	0.84	-0.08	
	CS12	36	7	0.61	0.57	0.58	-0.07	
	Cli100	34	4	0.32	0.36	0.37	0.11	
	CS08	29	24	0.86	0.93	0.95	0.07	*
Gulf of Carpentaria	LS15	57	6	0.19	0.18	0.18	-0.06	
	CT05	46	13	0.74	0.82	0.83	0.10	
	CS12	67	6	0.61	0.49	0.49	-0.25	*
	Cli100	61	6	0.25	0.29	0.30	0.17	
	CS08	48	30	0.85	0.95	0.96	0.10	

Key: Significant deviations from Hardy-Weinberg equilibrium are shown (*).

Population subdivision for *C. sorrah *was detected between Indonesia and the three Australian collection locations, but not within Australian waters, and the pattern of genetic population subdivision was similar for mtDNA control region sequences and microsatellite allele frequencies. *F*-statistics based on mtDNA ranged from 0.751 to 0.903 and for microsatellite data from 0.038 to 0.047 (Table 6).

**Table 6:** Statistically significant microsatellite pairwise F-statistics (below diagonal) and mtDNA Φ_ST _(above diagonal) for populations of *Carcharhinus sorrah*.

*C. sorrah*				
	West Australia	Indonesia	Gulf of Carpentaria	East Australia
West Australia	-	0.903	NS	NS
Indonesia	0.038	-	0.751	0.823
Gulf of Carpentaria	NS	0.047	-	NS
East Australia	NS	0.040	NS	-

Key: F-statistics were significant using an experiment wide alpha of 0.05 after adjusting for multiple comparisons via a sequential Bonferroni correction. NS indicates non-significance.

### Prionace glauca

**Figure 4:**
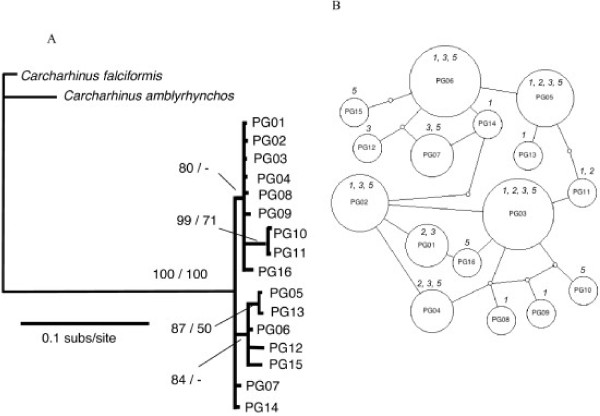
**Inferred phylogeny (A) and statistical parsimony network (B) among haplotypes of *P. glauca *collected in Indonesia (Ind) and Australia (Aust)**. The phylogeny was rooted with *C. falciformis *and *C. amblyrhynchos *and nodal support is given as Bayesian posterior probabilities/ML boostrap support. Dash (-) indicates support of less than 50%. In the network, each indicated step (circle) represents a single nucleotide difference in the mtDNA control region sequence. The area of circles is scaled to represent the relative frequency of that haplotype and the smallest circle represent inferred haplotypes that were not sampled. The collection location of sampled haplotypes is numbered (in italics) according to Fig. 1.

Sixteen mtDNA control region haplotypes were identified (Table 7) with little or no evidence of partitioning among populations. The most common haplotypes (PG03 and PG06) were evenly distributed across the four collection locations. Only the rare haplotypes; for example PG10, PG15 and PG16, were found in one geographic location; this apparent range restriction was because they were only sampled once each (Table 7). Haplotype and nucleotide diversity was similar across collection locations. There are no well-supported clades on the phylogenetic tree of haplotypes that show distinctiveness, and the haplotype network has no distinct structure (Fig. 4).

**Table 7:** Control region mtDNA haplotypes (with numbered polymorphic sites), haplotype frequencies, shared haplotypes and indices of population diversity for *Prionace glauca*.

*P. glauca *haplotypes (373 bases) Total N = 60																
Haplotype	2	5	8	9	1	1	1	2	2	2	2	2	Indonesia (n = 19)	Mid-north Pacific (n = 20)	West Australia (n = 4)	East Australia (n = 17)
	7	2	6	7	4	8	9	2	2	4	5	6				
					5	1	0	7	8	2	5	7				

PG01	C	G	A	C	G	T	G	C	T	G	T	T	0.105	-	0.250	-
PG02	.	.	.	.	.	.	.	T	.	.	.	.	0.158	0.150	-	0.176
PG03	T	.	.	.	.	.	.	T	.	.	.	.	0.211	0.250	0.250	0.118
PG04	.	.	.	.	.	C	.	T	.	.	.	.	0.105	0.100	0.250	-
PG05	T	.	.	G	.	.	.	T	.	.	A	C	0.105	0.050	0.250	0.188
PG06	.	.	.	G	.	.	.	T	.	.	A	C	0.211	0.150	-	0.294
PG07	.	A	.	G	.	.	.	T	.	.	.	C	0.053	0.150	-	-
PG08	T	.	.	.	.	C	.	T	C	.	.	.	-	-	-	0.059
PG09	T	A	.	.	.	C	A	T	.	.	.	.	-	-	-	0.059
PG10	T	A	.	.	A	.	.	T	.	.	.	.	-	0.050	-	-
PG11	T	.	.	.	.	.	.	T	.	.	A	.	-	-	-	0.059
PG12	.	A	G	G	.	.	.	T	.	.	A	C	0.053	-	-	-
PG13	T	.	.	G	.	.	.	T	.	A	A	C	-	-	-	0.059
PG14	.	.	.	G	.	.	.	T	.	.	.	C	-	-	-	0.059
PG15	.	C	.	.	.	.	.	T	.	.	A	C	-	0.050	-	-
																
PG16	T	.	.	.	.	.	.	.	.	.	.	.	-	0.050	-	-
Number of haplotypes													8	9	4	9
Number of polymorphic sites													8	8	6	9
Nucleotide diversity per location (within population, %)													0.740 ± 0.468	0.742 ± 0.458	0.878 ± 0.677	0.778 ± 481
Haplotype diversity per location (within population)													0.894 ± 0.037	0.895 ± 0.040	1.000 ± 0.177	0.890 ± 0.054

Key: Haplotype PG01 has Genbank accession number FJ161689.

*Prionace glauca *samples from four populations were assayed with five microsatellite loci. Sample sizes were adequate, except for the number of genotypes produced using locus Cli100 on samples from the Mid North Pacific (Table 8). Two populations (West Australia and East Australia) showed evidence of lack of equilibrium for genotype proportions for two loci, CS02 and CT06. Widespread disequilibrium in genotype proportions for locus CS02, which was reported here for *C. obscurus *samples, was not a feature of the results for *P. glauca*, except for samples from West Australia where null alleles were implicated. Detailed analyses of locus CT06 raw data suggested that allele stuttering might have resulted in scoring errors, as there was a significant shortage of heterozygote genotypes with alleles of one repeat unit difference. Instances of departures from Hardy-Weinberg proportions for *P. glauca *data were judged to be minor and unlikely to bias results. Unbiassed heterozygosity for *P. glauca *loci was 0.58 ± 0.05 and the number of alleles was 15.00 ± 1.08 for locus CS02, 5.00 for locus CT04, 5.75 ± 0.25 for locus CT06, 3.00 ± 0.41 for locus Cli107 and 4.25 ± 0.75 for locus Cli100 across loci and populations. Genotypic disequilibrium was not detected for pairwise comparisons of the five loci in the four populations.

**Table 8:** The population, sample size (N), number of microsatellite alleles per locus (Na), average observed heterozygosity (Ho) and expected (He) and unbiased (UHe) heterozygosity and fixation index (F) for each sampling location for *Prionace glauca*.

*P. glauca*								
	Locus	N	Na	Ho	He	UHe	F	Signif
Indonesia	CS02	26	15	0.81	0.83	0.85	0.03	
	CT04	33	5	0.55	0.63	0.64	0.13	
	CT06	30	6	0.40	0.57	0.58	0.30	
	Cli107	31	2	0.26	0.31	0.32	0.17	
	Cli100	28	5	0.36	0.37	0.37	0.02	
Mid North Pacific	CS02	20	17	0.95	0.88	0.90	-0.08	
	CT04	20	5	0.75	0.67	0.69	-0.11	
	CT06	20	6	0.55	0.62	0.64	0.11	
	Cli107	20	3	0.15	0.19	0.19	0.19	
	Cli100	3	2	0.00	0.44	0.53	-	
West Australia	CS02	21	12	0.62	0.83	0.85	0.26	*
	CT04	47	5	0.70	0.69	0.69	-0.02	
	CT06	43	6	0.35	0.61	0.62	0.43	*
	Cli107	42	4	0.31	0.36	0.37	0.15	
	Cli100	37	5	0.30	0.37	0.37	0.19	
East Australia	CS02	16	16	0.88	0.87	0.89	-0.01	
	CT04	18	5	0.56	0.64	0.66	0.13	
	CT06	17	5	0.41	0.66	0.68	0.38	*
	Cli107	14	3	0.29	0.36	0.37	0.20	
	Cli100	16	5	0.38	0.42	0.43	0.10	

Key: Significant deviations from Hardy-Weinberg equilibrium are shown (*). Heterozygous individuals were not detected among the three mid-north Pacific samples genotyped for locus Cli100.

There were no instances of genetic subdivision between collection locations for *P. glauca *from Australia, Indonesia or the northern Pacific Ocean with either class of genetic marker (Table 9).

**Table 9:** Statistically significant microsatellite pairwise F-statistics (below diagonal) and mtDNA Φ_ST _(above diagonal) for populations of *Prionace glauca*.

*P. glauca*				
	Indonesia	Mid North Pacific	West Australia	East Australia
Indonesia	-	NS	NS	NS
Mid North Pacific	NS	-	NS	NS
West Australia	NS	NS	-	NS
East Australia	NS	NS	NS	-

Key: F-statistics were significant using an experiment wide alpha of 0.05 after adjusting for multiple comparisons via a sequential Bonferroni correction. NS indicates non-significance.

### Sphyrna lewini

**Figure 5:**
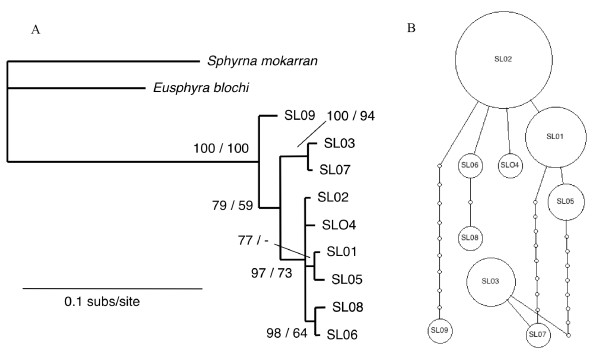
**Inferred phylogeny (A) and statistical parsimony network (B) among haplotypes of *S. lewini***. The phylogeny was rooted with *S. mokarran *and *E. blochi *and nodal support is given as Bayesian posterior probabilities/ML boostrap support. Dash (-) indicates support of less than 50%. In the network, each indicated step (circle) represents a single nucleotide difference in the mtDNA control region sequence. The area of circles is scaled to represent the relative frequency of that haplotype and the smallest circle represent inferred haplotypes that were not sampled. Haplotype SL09 from the North Atlantic (Atl) is equivalent to Duncan et al [15] haplotype number 16. The collection location of sampled haplotypes is numbered (in italics) according to Fig. 1.

Eight mtDNA control region haplotypes were identified amongst Indonesian and Australian samples (Table 10). Haplotype SL02 was the most common and was found in all collection locations at similar frequencies. As such it provided little to no evidence of population genetic structure on this geographic scale. Likewise, the distribution of remaining haplotypes provided no consistent information about gene flow between populations. An Atlantic Ocean haplotype [number 16, 15] was included here as SL09 to highlight the divergence between the Indo-Pacific and Atlantic Ocean areas. Haplotypes SL03 and SL07 formed a well-supported clade in the phylogeny and were outliers within the haplotype network (Fig. 5). Suprisingly, they were as distinct from the remainder as the Atlantic haplotype (SL09). They were sampled between one (SL07) and four times (SL03) from West Australia and Indonesia populations.

**Table 10:** Control region mtDNA haplotypes (with numbered polymorphic sites), haplotype frequencies, shared haplotypes and indices of population diversity for *Sphyrna lewini*.

*S. lewini *haplotypes (381 bases, '-' is an indel) Total N = 47																							
Haplotype	3	7	8	9	1	1	1	1	1	1	1	1	1	2	2	2	2	2	3	3	Indonesia (n = 28)	East Australia (n = 15)	West Australia (n = 4)
	0	6	3	5	1	1	2	2	2	3	3	4	6	5	8	8	9	9	5	5			
					5	7	0	1	3	8	9	7	8	1	1	3	8	9	1	2			

SL01	A	A	C	A	T	G	T	C	C	T	C	A	T	A	T	A	T	A	T	A	0.214	0.267	-
SL02	.	.	.	.	.	.	.	.	.	.	.	.	C	.	.	.	.	.	.	.	0.464	0.600	0.750
SL03	.	.	.	.	.	A	.	.	T	C	.	.	.	G	C	G	C	G	C	G	0.143	-	0.250
SL04	.	.	T	.	.	.	.	.	.	.	.	.	C	.	.	.	.	.	.	.	0.036	-	-
SL05	.	.	.	.	.	.	.	.	.	.	.	.	.	G	.	.	.	.	.	.	0.036	0.133	-
SL06	.	.	.	.	.	.	.	.	.	.	.	T	C	.	.	.	.	.	.	.	0.036	-	-
SL07	.	.	.	.	.	A	.	.	T	C	.	.	.	.	C	G	C	G	C	G	0.036	-	-
SL08	.	.	.	.	.	.	.	T	.	.	.	T	C	.	.	.	.	.	.	.	0.036	-	-
SL09	T	T	.	G	C	-	A	.	.	C	T	.	C	.	C	G	.	.	.	.	-	-	-
Number of haplotypes																					8	3	2
Number of polymorphic sites																					15	2	
Nucleotide diversity per site (within population, %)																					1.112 ± 0.635	0.204 ± 0.176	1.631 ± 0.117
Haplotype diversity per location (within population)																					0.738 ± 0.068	0.590 ± 0.106	0.500 ± 0.265

Key: Haplotype SL01 has Genbank accession number FJ161690. Haplotype SL09 represents an individual collected from the North Atlantic and has sequence that is equivalent to Duncan et al [15] haplotype number 16.

Sample sizes of genotypes assayed with microsatellite loci were adequate for *S. lewini*, with the mean (± SE) number being 52.78 (6.17) over the three collection locations and three microsatellite loci (Table 11). However, the degree of polymorphism of one locus (CT06) was poor. It was monomorphic in Indonesia and East Australia, had only two alleles in West Australia and heterozygotes were not detected. Lack of Hardy-Weinberg equilibrium was detected for locus Cli100 in Indonesia and locus CT07 in West Australia, which detailed analyses suggested was due to cryptic allelic stuttering or presence of null alleles. Genotypic disequilibrium was not detected for pairwise comparisons of the three loci in the three populations.

**Table 11:** The population, sample size (N), number of microsatellite alleles per locus (Na), average observed heterozygosity (Ho) and expected (He) and unbiased (UHe) heterozygosity and fixation index (F) for each sampling location for *Sphyrna lewini*.

*S. lewini*								
	Locus	N	Na	Ho	He	UHe	F	Signif
Indonesia	CT06	74	1		Monomorphic			
	CT07	74	6	0.66	0.71	0.71	0.06	
	Cli100	82	14	0.82	0.87	0.87	0.06	*
East Australia	CT06	44	1		Monomorphic			
	CT07	44	6	0.80	0.71	0.72	-0.12	
	Cli100	44	10	0.80	0.85	0.86	0.06	
West Australia	CT06	42	2	0.00	0.17	0.17	-	
	CT07	31	7	0.61	0.72	0.73	0.15	*
	Cli100	40	11	0.75	0.87	0.88	0.13	

Key: Significant deviations from Hardy-Weinberg equilibrium are shown (*).

As for *P. glauca*, genetic subdivision among *S. lewini *populations from Indonesia, East Australia and West Australia was not detected with mtDNA control region sequences or microsatellite allele frequencies (Table 12).

**Table 12:** Statistically significant microsatellite pairwise F-statistics (below diagonal) and mtDNA Φ_ST _(above diagonal) for populations of *Sphyrna lewini*.

*S. lewini*			
	Indonesia	East Australia	West Australia
Indonesia	-	NS	NS
East Australia	NS	-	NS
West Australia	NS	NS	-

Key: F-statistics were significant using an experiment wide alpha of 0.05 after adjusting for multiple comparisons via a sequential Bonferroni correction. NS indicates non-significance.

## Discussion

We have shown here that fisheries stocks of two species (*P. glauca *and *S. lewini*) most likely extend across the economic zones of Australia and Indonesia, suggesting that both countries are likely to be exploiting the same resource. Conversely, there is evidence of genetic population subdivision between the fisheries stocks of the spot-tail shark (*C. sorrah*) of the two countries, and there is some genetic evidence that a fourth species (dusky shark, *C. obscurus*) may also be subdivided along these lines. *Prionace glauca *and *S. lewini *are relatively large, ocean-going shark species, which would be expected to move freely across the Arafura and Timor Seas that are bisected by the fishing zones of the two countries.

While neither *C. sorrah *or *C. obscurus *are off-shore or truly pelagic species the finding of population subdivision was not expected. However, the robust design of this study adds weight to this finding. Two classes of independent genetic markers were used. Mitochondrial DNA is cytoplasmic, whereas microsatellite loci are derived from DNA in the cell nucleus. MtDNA is maternally inherited, while microsatellite loci are biparentally inherited [43]. Nucleotide sequencing was used for mtDNA, while variation in allelic frequencies was assessed for microsatellite loci.

Concordance between markers is taken as robust evidence of population structure [eg. [44,45]] and is considered best-practise when defining fisheries stocks [46]. The finding of genetic subdivsion between *C. sorrah *populations is emphasised by the apparent lack of subdivision among samples of other species (eg. *P. glauca *and *S. lewini*) that were collected from the same locations and analysed with the same suite of genetic methods. The genetic population subdivision between northern Australia and Indonesia reported here for *C. sorrah *needs to be tested with other methods, such as parasite distribution and abundance [eg. [11]] or stable isotope analysis of hard parts [eg. [47]].

The application of a range of non-genetic methods in further testing fisheries stock boundaries in all four shark species studied here would be valuable in view of the challenges faced in this study with the application of microsatellite markers. Cross-species, rather than species-specific, loci were used due to the limited availability of microsatellite loci for sharks and rays. This may have been responsible for comparatively inconsistent amplification of loci across samples within a species, leading to lower than ideal sample sizes. Other problems that may be potentially clarified in future by using species-specific loci may include Hardy-Weinberg disequilibrium and null alleles.

There are 31 species in the genus *Carcharhinus *[13]. The majority are found close to shore and are susceptible to exploitation. Nine globally distributed species (*C. albimarginatus, C. altimus, C. brevipinna, C. falciformis, C. leucas, C limbatus, C. longimanus, C. obscurus*, and *C. plumbeus*) occur in the Indo-Pacific, and the distribution of a further 14 species is centred on this region; *C. amblyrhynchoides *(also found in Indian Ocean), *C. amboinensis *(with Indian), *C. amblyrhynchos *(with west Pacific)*C. borneensis, C. cautus, C. dussumieri *(with Indian), *C. fitzroyensis, C. hemiodon *(with Indian), *C. macloti *(with Indian), *C. melanopterus *(with Indian and west Pacific), *C. sealei *(with Indian), *C. sorrah *(with Indian and west Pacific), and *C. tilstoni *and *Carcharhinus sp. A*. Population genetic subdivision has not been investigated for any of these species in the Indo-Pacific or elsewhere, except for *C. sorrah *(this study) and *C. limbatus *[48]. Keeney and Heist [48] found two major clades between ocean basins (western Atlantic, Gulf of Mexico, and Caribbean Sea clades, and eastern Atlantic, Indian, and Pacific Ocean clades) based on mtDNA control region sequence, and shallow population structure within ocean basins. We strongly recommend the assessment of population structure of other *Carcharhinus *species in the Indo-Pacific to assist with their conservation. The group of species has high regional biodiversity, economic importance and high levels of exploitation, which predisposes them to non-sustainable harvesting.

This is underlined by our finding that at least one Indo-Pacific species (*C. sorrah*) is potentially exposed to local population depletion. There is concern for the status of *C. sorrah *populations in Indonesia as fishing methods target this species, but the relative catch rate is consistently below that of the fishery in northern Australia [49]. Here we report that Indonesian and northern Australian populations are genetically subdivided. Consequently, the exploited Indonesian populations may not be replenished by migration, which justifies concerns for their susceptibility to overexploitation. As *C. sorrah *is commonly reported from inshore waters less than 200 m, the deep water of the Timor Trench (2 – 3000 m) is implicated as the isolating mechanism preventing gene flow between northern Australia and central Indonesia. The Timor Trench divides the northwestern edge of the Sahul continental shelf from the islands of central Indonesia. Deep water was also implicated in the stock structure of commercial snappers in this region [44,50]. The degree of population genetic subdivision in *C. sorrah *in northern Australia in this study was similar to the overall low levels of genetic subdivision reported in a previous allozyme study [data not shown, [51]]. We agree with Lavery and Shaklee [51] that the species should be managed as a single unit in Australia.

Our mtDNA sequence data suggested that genetic population subdivision might exist between *C. obscurus *Australia and Indonesia. We strongly recommend that this hypothesis is tested with further studies as our sampling strategy, particularly on the Australian west coast was sparse. However, Indonesian samples possessed two haplotypes (CO1 and CO2) that were not found in Australia, and there was evidence that those haplotypes had a common evolutionary history. The same conclusion would have been reached if samples from West Australia were grouped into two sampling locations rather than one. Unfortunately, the power of microsatellite data to resolve population structure in this species may have been compromised for the comparison between East Australia and Indonesia, as sample sizes were low, but sample sizes were adequate for the comparison between West Australia and Indonesia.

In contrast to the genetic differentiation between northern Australian and Indonesian populations for *C. sorrah *and possibly *C. obscurus*, no differentiation is reported here on this scale for two other shark species; *P. glauca *and *S. lewini*. Furthermore, the inclusion of a northern Pacific Ocean sample of *P. glauca *suggests that genetic homogeneity in this species extends over large geographic scales. Both *P. glauca *and *S. lewini *are distributed worldwide, have large body sizes and hence are likely to have high dispersal capacity. Duncan et al [15] confirmed this for *S. lewini*. They found little mtDNA control region sequence variaton among samples from in-shore nursery grounds within ocean basins. However, they did discover an upper limit to gene flow in females, as genetic subdivision was pronounced between Pacific, Indian and Atlantic Ocean basins. Duncan et al [15] also found that control region haplotype diversity was pronounced within the Indo-Pacific and our study confirms this. Indonesian and west Australian haplotypes SL03 and SL07 were as distinct from the majority of *S. lewini *haplotypes as SL09, which was sampled from the North Atlantic Ocean and which has the same sequence as haplotype 16 from Duncan et al [15]. *Prionace glauca *populations have not been previously assessed for degree of genetic subdivision. This study and the work by Duncan et al [15] on a similarly oceanic species (*S. lewini*) provides an hypothesis that can be tested in future studies: genetic homogeneity in *P. glauca *extends within, but not between, ocean basins. Further research that contributes to the sustainable management of this species is urgently needed. Clarke et al [52] used DNA-based and morphological species identification methods to show that *P. glauca *dominated (17%, by weight) dried shark fin auctioned at a Hong Kong market.

This study on shark population subdivision parallels previous research on red and gold snapper in northern Australia and Indonesia, which led to cooperative fisheries management actions directed at ensuring sustainability. Four Lutjanidae snapper species with similar biology were compared from the same collection locations. The species had the same habitat preferences (marine, reefs to 180 m) except for *L. argentimaculatus*, whose juveniles inhabit freshwater before becoming fully marine on maturation. Unlike shark species, snapper species are highly fecund (10^6 ^eggs/female/spawning season) with pelagic eggs and larvae. But like sharks, there was no expectation of population subdivision in the Indo-Pacific. One species (*Pristipomoides multidens*) had pronounced genetic subdivision [50,53]. The two red snapper species had moderate [*Lutjanus erythropterus*, [44]] and low [*Lutjanus malabaricus*, [44]] degrees of subdivision. The fourth species [*Lutjanus argentimaculatus*, [54]] was not genetically subdivided. The presence of genetic subdivision was explained by previously unrecognised site fidelity across life history stages. New knowledge about the geographic extent of *L. malabaricus *fisheries stocks in the region allowed the development of a biomass dynamic stock assessment model, which showed that current levels of harvesting were unsustainable. Australia and Indonesia are working together to co-manage shared stocks and fishing practises for snapper species have been modified [2]. It is expected that the work reported here will contribute to co-management actions on shared shark stocks.

Genetic analyses of population structure are an important component of fisheries management. When populations are genetically subdivided this information can be used to define the geographic boundaries of a fisheries stocks. Boundaries provide the confidence to use estimates of recruitment, growth and mortality (natural and fishing) to describe fisheries populations in mathematical models. However, when genetic analyses of population structure suggest the absence of genetic subdivision, this implies fisheries stocks are widely distributed. If this occurs, two courses of action are appropriate. Firstly, further research should be sponsored to test the hypothesis of no subdivision using a range of genetic and non-genetic methods. Researchers need to keep management agencies up-to-date with latest results, in case stock subdivision is found and management can occur independently. Secondly, management arrangements need to be applied and coordinated among authorities responsible. It is not sufficient for exploitation in only one part of the stock to be regulated, as unregulated exploitation elsewhere in the stock could cause uniform depletion across the entire stock. Thus agencies across Australian states (eg. Queensland, Northern Territory and Western Australia) should cooperate to manage *C. sorrah*. Likewise, Australian and Indonesian management agencies should cooperate to manage Indo-Pacific *P. glauca *and *S. lewini *populations.

## Conclusion

Using mtDNA and microsatellite loci, this study has contributed to the definition of stock boundaries for one (*C. sorrah*) and maybe two (*C. obscurus*) exploited shark species, and has shown that the nations of Australia and Indonesia are most likely exploiting the same stocks of *P. glauca *and *S. lewini*. Australian authorities are aware of the need for catch limits on *C. sorrah *and Indonesia would be wise to take similar action. Indonesia is drafting a national plan of action for shark exploitation. Generally, we have also demonstrated the difficulties associated with predicting the presence of genetic subdivision even for co-distributed and ecologically similar species. For sustainable management and conservation purposes, we advocate a species-by-species assessment of population structure, at least until clear patterns emerge in the bio-diverse Indo-Pacific region.

## Authors' contributions

JO and JS developed, designed and coordinated the study. JO wrote and revised the manuscript and performed statistical analyses. TK carried out mtDNA sequence alignments and statistical analyses. DB and JG participated in data analyses and interpretation. All authors edited the manuscript drafts and approved the final version.
